# The Neuro-Immuno-Senescence Integrative Model (NISIM) on the Negative Association Between Parasympathetic Activity and Cellular Senescence

**DOI:** 10.3389/fnins.2018.00726

**Published:** 2018-10-09

**Authors:** Torvald F. Ask, Ricardo G. Lugo, Stefan Sütterlin

**Affiliations:** ^1^Research Group on Cognition, Health, and Performance, Institute of Psychology, Inland Norway University of Applied Sciences, Lillehammer, Norway; ^2^Faculty of Health and Welfare Sciences, Østfold University College, Halden, Norway; ^3^Division of Clinical Neuroscience, Oslo University Hospital, Oslo, Norway

**Keywords:** heart rate variability, cytokines, telomere damage, NFkB, cellular senescence, reactive oxygen species, parasympathetic activity, the neuro-immuno-senescence integrative model

## Abstract

There is evidence that accumulated senescent cells drive age-related pathologies, but the antecedents to the cellular stressors that induce senescence remain poorly understood. Previous research suggests that there is a relationship between shorter telomere length, an antecedent to cellular senescence, and psychological stress. Existing models do not sufficiently account for the specific pathways from which psychological stress regulation is converted into production of reactive oxygen species. We propose the neuro-immuno-senescence integrative model (NISIM) suggesting how vagally mediated heart rate variability (HRV) might be related to cellular senescence. Prefrontally modulated, and vagally mediated cortical influences on the autonomic nervous system, expressed as HRV, affects the immune system by adrenergic stimulation and cholinergic inhibition of cytokine production in macrophages and neutrophils. Previous findings indicate that low HRV is associated with increased production of the pro-inflammatory cytokines IL-6 and TNF-α. IL-6 and TNF-α can activate the NFκB pathway, increasing production of reactive oxygen species that can cause DNA damage. Vagally mediated HRV has been related to an individual’s ability to regulate stress, and is lower in people with shorter telomeres. Based on these previous findings, the NISIM suggest that the main pathway from psychological stress to individual differences in oxidative telomere damage originates in the neuroanatomical components that modulate HRV, and culminates in the cytokine-induced activation of NFκB. Accumulated senescent cells in the brain is hypothesized to promote age-related neurodegenerative disease, and previous reports suggest an association between low HRV and onset of Alzheimer’s and Parkinson’s disease. Accumulating senescent cells in peripheral tissues secreting senescence-associated secretory phenotype factors can alter tissue structure and function which can induce cancer and promote tumor growth and metastasis in old age, and previous research suggested that ability to regulate psychological stress has a negative association with cancer onset. We therefore conclude that the NISIM can account for a large proportion of the individual differences in the psychological stress-related antecedents to cellular senescence, and suggest that it can be useful in providing a dynamic framework for understanding the pathways by which psychological stress induce pathologies in old age.

## Introduction

In this paper, we propose the neuro-immuno-senescence integrative model (NISIM), which is a mechanistic model for prefrontally modulated vagal activity that accounts for the cognitive-affective and psychosocial modulators of the stress response, and the subsequent physiological pathways to the oxidative cellular stresses that antecedes cellular senescence. Cellular senescence is a tumor-suppressive mechanism in which stressed cells stop progressing through cell-cycle (Wright and Shay, 2001; Dimri, 2005) and arrest growth (Campisi, 2013). Potential stressors include oxidative stress, DNA damage, activation of tumor-suppressive pathways, mitogens and proliferation-associated signals. Depending on the stressor, certain proteins capable of sensing the stressor get activated and initiate the response by changing the genetic expression of the cell (d’Adda di Fagagna et al., 2003; Takai et al., 2003; Herbig et al., 2004). For instance, DNA damage will activate a DNA damage response (DDR) protein that arrest cell division through activation of the p53 and p16-pRB tumor suppressive pathways (Olsen et al., 2002; Michaloglou et al., 2005). Being a tumor-suppressive mechanism, cellular senescence primarily happens in mitotic cells from which cancer can develop (DiLeonardo et al., 1994; Chen et al., 2000). Thus, cellular senescence blocks cellular-level tumor formation. Mutations in the TP53 gene, which is a gene that regulates p53-dependent growth arrest, occurs in close to all types of cancer at rates varying between 10 and 100% (Rivlin et al., 2011).

Senescent cells are resistant to apoptosis and tend to accumulate with age (Dimri et al., 1995; Tepper et al., 2000; Hampel et al., 2004). They also secrete a wide range of bioactive molecules (SASP factors) as part of the senescence-associated secretory phenotype (SASP; Tchkonia et al., 2013). SASP factors have both positive and negative effects on tissues. In addition to prevent cancer and promote wound healing, evidence also indicate that they alter tissue microenvironments over time by creating inflammatory milieus (Tchkonia et al., 2013). Because senescent cells secrete SASP factors that cause inflammation, and because they accumulate with age, researchers believe that they are drivers of many age-related pathologies, such as neurodegenerative diseases (Baker D.J. et al., 2011; Chinta et al., 2015) and cancer (Bissel et al., 2005; Rao and Jackson, 2016) which is contradictory to its protective and tumor-suppressive effects.

Evolutionary explanations have been proposed to clarify how cellular senescence can be both tumor-suppressive and tumorigenic. Tissue renewal is essential for complex organisms to maintain longevity. Organisms with renewable tissues are susceptible to developing cancer due to cellular mutations (Hanahan and Weinberg, 2000), and mitotic cells acquire mutations more frequently than non-mitotic cells (Busuttil et al., 2006). This have ushered the evolution of tumor-suppressive mechanisms such as cellular senescence that are capable of halting uncontrolled cell division (Sager, 1991; Braig and Schmitt, 2006). Due to extrinsic hazards, organisms did not live long in the environments that this mechanism evolved in, meaning that cellular senescence would be effective over a shorter duration than it is today. With today’s unprecedented high life expectancy, senescent cells can accumulate, making cellular senescence more beneficial to young organisms than older ones (Kirkwood and Austad, 2000). Cellular senescence is therefore linked with health span, and consequently, lifespan (Baker et al., 2015). Tumor growth (Mouton et al., 2011; Guo et al., 2015) and other factors related to senescence, such as telomere length (Kroenke et al., 2011), and health-/lifespan (Piccirillo et al., 1998; Shimizu et al., 2002) are related to parasympathetic activation. Parasympathetic activation and its central nervous modulation is key to psycho-physiological adaptability and can be indexed by peripheral proxies such as baroreflex sensitivity or vagally mediated heart rate variability (HRV), which is regulated by the central-autonomic-network (CAN). The NISIM attempts to provide a plausible, mechanistic explanation for how psycho-physiological adaptability relates to the molecular antecedents to telomere attrition and consequently, cellular senescence.

### Vagally Mediated Heart Rate Variability: The Central-Autonomic-Network and Stress Regulation

Vagally mediated HRV is a well-established biomarker for an individual’s capability to adapt to, and thus regulate acute and chronic stress (Appelhans and Luecken, 2006; Thayer et al., 2012) by the modulation of physiological arousal (Gross, 1998). The autonomic nervous system (ANS) innervates the heart with parasympathetic (PNS) and sympathetic (SNS) branches. The SNS, mediated by neurotransmission of nor-adrenaline, has an excitatory influence on heart rate, while the PNS, mediated by acetylcholine neurotransmission, has inhibitory influence on heart rate. The peak effect of acetylcholine arrives faster than the peak effect of noradrenaline, consequently leading the oscillations in heart rate produced by the SNS and the PNS to occur at different speeds (Berntson et al., 1997). Increased heart rate could arise from increased SNS activity or decreased PNS activity (vagal withdrawal), although research on conscious animals indicate that these processes occur concomitantly, with sympathetic positive feedback reflexes of the mechanical stretch of the aorta inhibiting arterial baroreflex sensitivity consequently reducing vagal influences on heart rate (Pagani et al., 1982). PNS inhibition remains dominant at rest and maintains a resting heart rate that is below the intrinsic firing rate of the heart. Thus, an individual’s capacity to rapidly adapt its arousal states depends in large on the PNS’ contribution (Berntson et al., 1997).

The autonomic influences on heart rate are remotely regulated by the network of brain areas constituting the CAN (Benarroch, 1993). The medial prefrontal cortex (mPFC) dominates the regulatory output of the CAN due to its descending connections to pre-autonomic cell groups in the hypothalamus, periaqueductal gray, and brainstem (Seeley et al., 2007). According to the neurovisceral integration model (Thayer and Lane, 2000; Thayer et al., 2012), higher prefrontal cortical activation is associated with a higher capacity for adaptive behavioral and emotional responding and subsequent return to homeostasis after ceased stress exposure. Higher variation between consecutive heart beats are due to greater PNS input via vagally mediated, and prefrontally modulated cortical influences. The PNS inhibitory influence on heart rate is considered a ‘vagal brake’ on initial and automatic physiological responses, which is how it is thought to facilitate an adaptation to environmental stressors (Porges, 1997, 2001).

Mental stress is associated with neural-SNS modulation of cardiac activity consequently reducing HRV (Pagani et al., 1989); pure autonomic failure characterized by the degeneration of post-ganglionic sympathetic nerve fibers is associated with reductions in total HRV (Furlan et al., 1995). However, giving a detailed description of role of the SNS in HRV is outside the scope of this article.

Coping with stress depends on coping strategies such as cognitive reappraisal, distraction, or others. These strategies rely heavily on the functioning of prefrontal cortical structures. Thus, the activation levels in the PFC are associated with the demand on regulatory efforts, i.e., the level of experienced stress (Golkar et al., 2012). Higher levels of psychological stress, marked by higher activations in limbic structures and increased heart rate, require higher levels of prefrontal activation and effortful processing (Golkar et al., 2012). The prefrontal cortex can reduce stress arousal by: (1) frontal inhibition of the limbic system, reducing excitatory impulses from the amygdala to the SNS, or; (2) via increased stimulation of the vagus nerve, which counters the arousing effects of the SNS. Both modes of stress reduction will be reflected in reduced heart rate and a relative PNS dominance on cardiac innervation, thus causing increased HRV. Lower prefrontal activation has the possible consequence of not being able to match the neurophysiological demands of the psychological stressor, resulting in less efficient PNS inhibitory influences on the heart, and subsequently, prolonged stress arousal marked by perpetuations of constant, elevated high heart rate. At rest, the prefrontal cortex keeps the amygdala under frontal inhibitory tonic control, with lower prefrontal activation leading to disinhibition of the amygdala, reflected in greater fluctuations in psychological stress arousal (Thayer and Lane, 2009). Thus, being an indicator of PNS activity and a proxy for prefrontal activation, higher vagally mediated HRV is related to fine-grained control over arousal during prolonged stressors (Hildebrandt et al., 2016) expressed as a stress resilient mode of coping and recovery. It is important to note, however, that although vagally mediated HRV is used as an index for prefrontal modulation of vagally activity, it is a marker that reflects vagal modulation of cardiac activity, hence, generalizations to overall PFC, PNS, and ANS functioning should be interpreted with caution.

Studies conducted over the past decade have indicated links between psychological stress and antecedents to cellular senescence (Epel et al., 2004; Mathur et al., 2016), however, current psycho-physiological models (Sapolsky, 2004; Epel et al., 2009) do not sufficiently explain how these phenomena are related. An early model proposed that the perception of chronic stressors is modulated by psychosocial factors and personality, which if not giving ways to adequate modes of coping will chronically activate the stress response, marked by increased SNS activity and subsequent release of glucocorticoids (Sapolsky, 2004). The chronic activation is followed by a “series of as yet unknown steps” (Sapolsky, 2004, p. 17323) that subsequently lead to increased production of reactive oxygen species and oxidative damage to telomeres. In a review of various stress-related cognitions and possible endocrine correlates, a model that considered the opposing effects of threat cognitions and mindfulness on cellular aging was proposed (Epel et al., 2009). In the model proposed by Epel and colleagues, cognitions such as threat appraisal and ruminative thoughts prolong states of reactivity (Lazarus and Folkman, 1984), attributing telomere damage to high cortisol (Epel et al., 2006), insulin insensitivity (Gardner et al., 2005), and oxidative stress (Liu and Mori, 1999; Sampson et al., 2006), while mindfulness meditation techniques shift threat appraisal to challenge appraisal (Lazarus and Folkman, 1984; Epel et al., 2009), reduce rumination (Teasdale et al., 1995) and stress arousal (MacLean et al., 1997; Infante et al., 1998; Schneider et al., 2005; Paul-Labrador et al., 2006), and in turn increase positive arousal (Glaser et al., 1992; Walton et al., 1995; Wolkowitz et al., 2001), attributing telomere integrity to high androgens (Bernhardt et al., 1998; Chichinadze and Chichinadze, 2008; Mendes et al., 2008), high growth hormone axis activity (Kiaris and Schally, 1999; Akiyama et al., 2002; Torella et al., 2004), and increased vagal tone (Epel et al., 2006; Paul-Labrador et al., 2006; Gramzow et al., 2008). In addition to the lack of specific processes, the common denominator for both Sapolsky (2004) and Epel et al. (2009) models are that they are broad and general in the way they explain the psycho-physiological antecedents to cellular senescence. The NISIM is based on the assumption that prefrontally modulated vagal activity can encompass a large proportion of the personality and psychosocial modifiers in Sapolsky (2004) model and that measuring vagally mediated HRV indicates how these modifiers affect activation of the stress response. The NISIM also propose that the prefrontal modulation of vagal activation can account for some of the physiological steps that are left unspecified in Sapolsky’s model, which suggests a more central role for vagal tone in cellular senescence than that proposed by Epel et al. (2009). Several mechanisms have been proposed for how psychological stress could be converted into cellular stress (Hayashi, 2014), including the production of pro-inflammatory cytokines and reactive oxygen species, both antecedents to events that can initiate the senescence response (Campisi, 2001, 2003). Regulation of psychological stress could therefore influence cellular senescence by affecting the production of pro-inflammatory cytokines and reactive oxygen species.

### Vagally Mediated HRV in Senescence-Related Pathways

**FIGURE 1:**
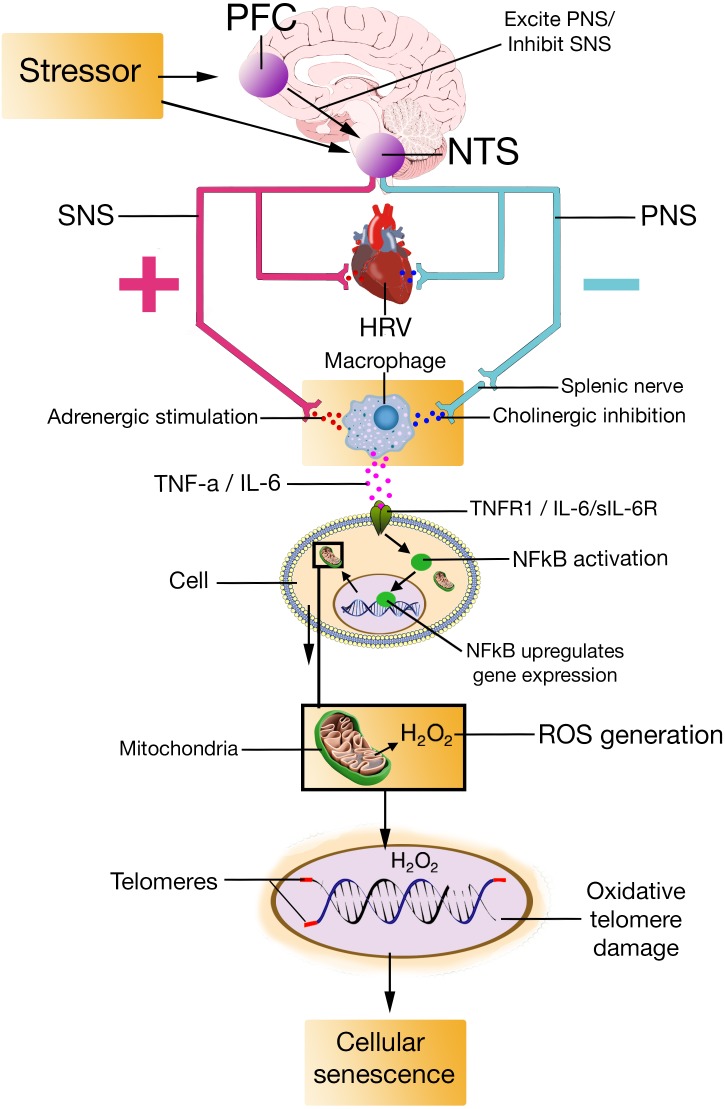
PFC, prefrontal cortex; NTS, nucleus of the tractus solitarius; SNS, sympathetic nervous system; PNS, parasympathetic nervous system; HRV, heart rate variability; TNF-a, Tumor Necrosis Factor-alpha; IL-6, interleukin-6; TNFR1, Tumor Necrosis Factor-alpha Receptor 1; sIL-6R, soluble interleukin-6 receptor; NFkB, Nuclear factor kappa light chain enhancer of activated B cells; H2O2, hydrogen peroxide; ROS, reactive oxygen species.

The mechanisms the NISIM propose are as follows (**Figure 1**): Prefrontally modulated and vagally mediated cortico-cardiac control indicated by quantitative measures of HRV is associated with an individual’s stress regulation capacity (Thayer and Lane, 2000; Thayer et al., 2012). Prefrontally modulated influences on the ANS affects the activity of the SNS and PNS fibers. Activity in SNS fibers descending into lymphoid tissues result in increased transmission of adrenergic ligands onto the adrenoceptors on macrophages, subsequently increasing cytokine production (Madden et al., 1995; Vizi, 1998). Activity in vagal fibers results in increased transmission of cholinergic ligands onto the cholinergic receptors on macrophages, mediated by the splenic nerve, subsequently reducing cytokine production (Wang H. et al., 2003; Rosas-Ballina et al., 2008). Individuals with lower stress regulation capacity have an increased adrenergic stimulation of macrophages which increases production and release of cytokines (Sloan et al., 2007; Woody et al., 2017a) that trigger the nuclear factor kappa light chain enhancer of activated B cells (NFκB) pathway (Sakurai et al., 2003; Feng et al., 2017) and cause an increase in reactive oxygen species (Schulze-Osthoff et al., 1992; Behrens et al., 2008). Connecting psychological stress regulation to production of reactive oxygen species via this pathway from the modulatory activity of the CAN to cytokine induced NFκB activation is, to our knowledge, novel. Increased reactive oxygen species due to reduced capacity to regulate psychological stress cause oxidative telomere damage (Zalli et al., 2013; Streltsova et al., 2017) resulting in cellular senescence (Campisi, 2001, 2003). We suggest that the novel contribution of the NISIM is the organization of these specific physiological mechanisms into a causal pathway that connects stress regulation, quantified by HRV, to cellular senescence. Considering the role of prefrontally modulated vagal activation can improve our understanding of the observed but still insufficiently explained indirect relationship between stress regulation and age-related disease. The following mechanisms are proposed for neurodegenerative diseases in relation to psychological stress: Peripheral cytokines produced because of low stress regulation capacity crosses the blood-brain barrier (Maletic and Raison, 2014), where they induce senescence in astrocytes and microglia (Mander et al., 2006; Jiang and Cadenas, 2014). Senescent glial cells start secreting SASP factors, altering tissue microenvironments, driving age-related neurodegenerative disease (Wang et al., 2012; Chinta et al., 2015). Similar pathways are proposed for psychological stress regulation and cancer, where cytokine levels produced because of low stress regulation capacity induces cellular senescence. Senescent cells secrete SASP factors and cause loss of tissue structure and function (Chinta et al., 2015) which induce cancer and tumor growth and metastasis over time (Campisi, 2013; Tchkonia et al., 2013).

A review of the literature on the interactions between the ANS and the immune system will be provided to outline a rationale for how prefrontally modulated vagal activity could be related to cytokine production, along with current evidence regarding the relationship between vagally mediated HRV and serum levels of cytokines. Evidence pertinent to the subsequent molecular pathways by which these cytokines increase production of reactive oxygen species that damage DNA will be discussed to elucidate the mechanisms underlying recent observations on the relationship between telomere length and vagally mediated HRV, and the effect of stress relaxation techniques on telomere length and telomerase activity. This will culminate in a further review of the cellular consequences of telomere dysfunction and subsequent initiation of the senescence response, along with the phenotypic profile of cells that senesce in response to telomere damage. These relationships and subsequent line of argumentation is what that the NISIM is based on. Findings apposite to how the senescence-associated secretory phenotype drive neurodegenerative disease and cancer will be expounded to provide a plausible explanation for recent evidence linking vagally mediated HRV, thus regulation of psychological stress, to onset of Parkinson’s disease, Alzheimer’s disease, and cancer. In a final discussion, predictions emanating from the model will be followed by critical appraisal of the reviewed evidence with regards to possible limitations and prospects.

## Stress and the Immune System

Pro-inflammatory cytokines, in addition to being implicated as a mechanism in the pathway from psychological to cellular stress (Hayashi, 2014) also initiate and maintain cellular senescence (Ren et al., 2009). These protein ligands are usually classified as either pro-inflammatory or anti-inflammatory depending on how they regulate the effects of the immune system. Two immune cells that are major sources of pro-inflammatory cytokines are the neutrophil and the macrophage. The pro-inflammatory cytokines that macrophages produce include interleukin (IL)-1, IL-6, IL-8 and tumor necrosis factor alpha (TNF-α; Oh et al., 1999; Dorner et al., 2002; Müller et al., 2003). Neutrophils secrete the pro-inflammatory cytokines IL-1β, IL-6, and TNF-α and IL-8 (Kasama et al., 2005; Tecchio et al., 2014; Naegelen et al., 2015). Understanding how vagal activity indexed by vagally mediated HRV is related to cytokine production warrants an explanation for how stress affects the immune system.

### Autonomic-Innate Immune System Activation

Central stress arousal can activate the immune system in several ways (Ader et al., 2001; Segerstrom and Miller, 2004). One such way is via the SNS fibers descending from the brain into lymphoid tissues like bone marrow, thymus, spleen, and lymph nodes (Felten and Felten, 1994). These fibers can transmit adrenergic ligands onto the α-subtype adrenoceptors of white blood cells that reside in these tissues, inducing immune responses such as increased cytokine production (Madden et al., 1995; Vizi, 1998). Furthermore, in a recent landmark paper, the neuro-innate signaling between the sympathetic nervous system and macrophages that controls catecholamine catabolism was implicated in the increased lipolysis driven by age-associated inflammation (Camell et al., 2017). In addition to inducing immune activity, the ANS is also capable of suppressing immune activity and inflammation (Tracey, 2002, 2007). This is achieved through a serially connected neural pathway from the vagus nerve to the immune system, with preganglionic origins in the dorsal motor nucleus of the vagus nerve, and postganglionic origins in the celiac-superior mesenteric plexus that projects in the splenic nerve (Rosas-Ballina et al., 2008; Pavlov and Tracey, 2012). This is called the inflammatory-reflex or immuno-reflex. The anatomical validity of this pathway was challenged by Nance and Sanders (2007), but was later verified by Rosas-Ballina et al. (2008). The immuno-reflex is mediated by cholinergic signaling, which interacts with the α7-subunit of the nicotinic acetylcholine receptor (nAChR) on macrophages and decreases TNF-α production (Wang H. et al., 2003; Huston et al., 2006) as well as IL-1β, IL-6, and IL-8 (Wang et al., 2004). Stimulation of the α7-subunit of the nAChR on neutrophils inhibits neutrophil recruitment and neutrophil secretion of TNF-α, removal of the receptor increases levels of TNF-α (Giebelen et al., 2007; Su et al., 2009). Vagotomy has been shown to increase serum and liver concentrations of TNF-α, electrical stimulation of the vagal nerve attenuates cytokine levels (Pavlov et al., 2003), and parasympathetic dysfunction is independently associated with neutrophil-lymphocyte ratio and C-reactive protein (CRP) levels (Ackland et al., 2018). This implicates the ANS, and more specifically, the vagus nerve in controlling the body’s systemic response to inflammation (Papaioannou et al., 2013), and demonstrates how the interplay between SNS activation and PNS inhibition of the immune response might be reflected in vagally mediated HRV, which is what the NISIM is based on.

### Cytokines and Vagally Mediated Heart Rate Variability in Healthy Participants

Psychological stress is known to promote increases in levels of IL-1, IL-6 and TNF-α (Fedorova and Sarafian, 2012; Gu et al., 2012). Several studies links both chronic and acute psychological stress to increases in circulating TNF-α and IL-6 (Cohen et al., 1999; Katsuura et al., 2012). An individual’s neurophysiological capacity to regulate arousal states, as indexed by HRV, should therefore play part in the degree to which the SNS and PNS induce and suppress immune activity, although the evidence is somewhat mixed.

Low vagal tone indicated by vagally mediated HRV was associated with elevated plasma levels of IL-6 and soluble tissue factor in healthy middle-aged men and women (von Känel et al., 2007), and in a study of healthy subjects from a sub-sample of the Coronary Artery Risk Development in Young Adults (CARDIA) study, vagally mediated HRV was strongly and inversely related to IL-6 and CRP levels (Sloan et al., 2007). Similarly, in a study of acute mental stress in healthy male participants, those with low vagal tone indicated by vagally mediated HRV demonstrated delayed recovery of TNF-α, diastolic blood pressure, and cortisol, for up to an hour after the stressor had ended (Weber et al., 2010).

In an earlier study of middle-aged, healthy men and women, also a sub-sample from the CARDIA study, Owen and Steptoe (2003) found increases in natural killer (NK) cell counts and the pro-inflammatory cytokines IL-6 and TNF-α in plasma samples drawn after a mental stressor task. The increases in NK cell and cytokines were positively associated with heart rate, but the study found no relationship between immune responses and vagally mediated HRV, either at rest or following stress. In concordance with other findings, Lampert et al. (2008) found that higher levels of inflammation in otherwise healthy middle-aged male twins was associated with autonomic dysregulation indicated by an inverse relationship between both vagally and sympathetically mediated HRV parameters and plasma levels of CRP, and IL-6. After adjusting for age and CAD risk factors, sympatho-vagally mediated HRV remained a significant predictor of CRP. A recent study of 30 young healthy women reported that low vagal tone indicated by reductions in vagally mediated HRV was associated with greater increase in TNF-α and IL-6 but not CRP in response to a speech stressor task (Woody et al., 2017a).

Albeit mixed (Owen and Steptoe, 2003; Lampert et al., 2008), the evidence seems to indicate that prefrontally modulated vagal activity, indicated by vagally mediated HRV is associated with serum levels of pro-inflammatory cytokines (Sloan et al., 2007; von Känel et al., 2007; Weber et al., 2010; Woody et al., 2017a), which according to the NISIM is due to lower prefrontal stress regulation capacity resulting in less cholinergic inhibition of inflammatory cytokine production, which in turn results in increased production of TNF-α and IL-6.

### TNF-α, IL-6 and the NFκB Pathway

TNF-α and IL-6 exert their effects in part by activating the nuclear factor kappa light-chain-enhancer of activated B cells (NFκB) pathway (Sakurai et al., 2003; Wang L. et al., 2003; Ma et al., 2010). NFκB is a rapid-acting transcription factor with receptor activity (Yamamoto and Gaynor, 2004; Jiang and Cadenas, 2014) and has a role in regulating cellular activities such as mitochondrial oxidative phosphorylation and adaptive metabolism (Mauro et al., 2011), and regulating inflammatory gene expression (Baker R.G. et al., 2011; Guma et al., 2011). In unstimulated cells, NFκB is localized in the cytoplasm and associated with a family of proteins called inhibitor of kB (IkB). When IkB is phosphorylated by IkB kinases (IKK) it causes the IkB to degrade and NFkB is subsequently released and translocates to the cell nuclei where it binds to DNA sites (called kB sites) and activate specific target gene expression (Yamamoto and Gaynor, 2004; Gilmore, 2006). TNF-α induced activation of the NFκB is the most extensively characterized signaling pathway for NFκB. When TNF-α binds to TNF-α receptor 1 (TNFR1), it immediately triggers assembly of signaling molecules on the intracellular domain of the receptor, which recruits IKK to the receptor associated proteins, and subsequent phosphorylation of IkB occurs (Sakurai et al., 2003; Yamamoto and Gaynor, 2004; Jiang and Cadenas, 2014). NFκB is expressed in all cells that react to cytokine stimulation (Gilmore, 2006). All tissues in the human body express TNFR1 (Fujita and Srinivasula, 2009). Only a few cell types express the membrane bound IL-6 receptor (mbIL-6R) on their cell surface, but a soluble form of the IL-6R (sIL-6R) has been detected in several body fluids, like blood and urine. sIL-6R has an affinity for IL-6 that matches that of the mbIL-6R, and the IL-6/sIL-6R complex interacts with a membrane protein called gp130 that is expressed throughout human tissue. The IL-6/sIL-6R activation of gp130 therefore represents a trans-signaling pathway alternative to the classic IL-6 pathway (Su et al., 2017). The IL-6/sIL-6R complex has been found to modulate NFκB activation (Feng et al., 2017). NFκB activity has been linked to psychosocial stress (Bierhaus et al., 2004), and both vagus nerve stimulation and acetylcholine reduce nuclear translocation of NFκB (Sun et al., 2013). Based on these observations, the NISIM suggests that for individuals with low prefrontally modulated vagal activity, indicated by low vagally mediated HRV, the increased production of cytokines due to less cholinergic stimulation of macrophages results in an increased activation of NFκB.

The regulation of genetic expression that results from NFκB activation has several immediate effects on cellular activity (Guma et al., 2011; Mauro et al., 2011), some of which include increased production of reactive oxygen species (ROS; Schreck and Bauerle, 1994; Mauro et al., 2011). Albeit having several important functions, excess ROS production lead to oxidative stress (OS; Halliwell, 1994; Betteridge, 2000; Imlay, 2003), which is a known antecedent to cellular senescence (Campisi, 2013).

## TNF-α and IL-6 Induced NFκB Regulated Production of Reactive Oxygen Species

The main sources for intracellularly generated ROS includes the mitochondria, peroxisomes, endoplasmic reticulum, and nicotinamide adenine dinucleotide phosphate oxidase (NOX; Karlsson and Dahlgren, 2002; Dunn et al., 2015), all of which are linked to TNF-α signaling (Beier et al., 1997; Yang et al., 2006; Jiang and Cadenas, 2014; Fischer and Maier, 2015). Several cytokines, including TNF-α and IL-6, are known to increase ROS production in various tissues (Corda et al., 2001; Yang et al., 2007; Behrens et al., 2008). TNF-α stimulates production of peroxides, particularly hydrogen peroxide (H_2_O_2_; Schulze-Osthoff et al., 1992; Schreck and Bauerle, 1994), as well as superoxide anions ($O_{2}^{-}$; Shoji et al., 1995) and has been found to increase intracellular ROS by 11,2% to 48,4%, and extracellular ROS by 142% to 680% in retinal pigment epithelial (RPE) cells, which is above levels induced by other inflammatory cytokines such as IL-1β and interferon gamma (Yang et al., 2007). IL-6 stimulate production of superoxides such as $O_{2}^{-}$ (Behrens et al., 2008). TNF-α and IL-6 increase ROS production in part by activating the NFκB pathway (Sakurai et al., 2003; Wang L. et al., 2003; Ma et al., 2010). Activation of NFκB causes a rapid and transient increase in H_2_O_2_ and $O_{2}^{-}$ (Schreck and Bauerle, 1994). Thus, individuals with low prefrontally modulated and vagally mediated HRV, and therefore less cholinergic inhibition of cytokine production by macrophages, will have an increased cytokine-induced activation of NFκB, and in turn, increased ROS production. H_2_O_2_ and $O_{2}^{-}$ molecules are capable of oxidizing biomolecules where oxygen itself reacts poorly (Imlay, 2003). This initiate chain reactions that can damage macromolecules such as DNA (Betteridge, 2000; Stadtman and Levine, 2003).

### The Role of TNF-α and IL-6 in ROS-Induced DNA Damage

Most DNA damage in human cells is caused by ROS-induced oxidative damage. ROS like $O_{2}^{-}$ and H_2_O_2_ cause damage by altering DNA bases to species such as 8-hydroxyguanine and 8-hydroxy-2′-deoxyguanosine (8-OH-dGua; Kasai et al., 2008; Valavanidis et al., 2009; Coluzzi et al., 2014), and if not repaired correctly, it causes both single strand and double strand breaks (Coluzzi et al., 2014). Several lines of evidence suggest that TNF-α induces oxidative DNA damage by mitochondria-produced H_2_O_2_ and $O_{2}^{-}$ (Fehsel et al., 1991; Shoji et al., 1995) which is how it relates to NFκB activity.

H_2_O_2_ damaged DNA triggers a cell death signaling via a pathway called the mitogen activated protein kinase (MAPK) Jun N-terminal kinase (JNK; MAPK JNK) pathway, a mechanism for apoptotic prevention of gene mutations and further damage (Dhanasekaran and Reddy, 2008). IL-6, in addition to causing ROS generation (Behrens et al., 2008), have also been shown to inhibit H_2_O_2_-induced apoptosis by upregulating proteins that blocks the JNK pathway. Cells with IL-6 exposure also fail to reduce peroxides generated by H_2_O_2_ (Lin et al., 2001). Furthermore, in IL-6 treated cells, only 50% of 8-OH-dGua is repaired after oxidative damage. This indicates that IL-6 can enhance cells susceptibility to H_2_O_2_-induced DNA damage (Lin et al., 2001).

The specific cytokines that are associated with low vagal tone indicated by vagally mediated HRV due to reduced cholinergic stimulation of macrophages, are also associated with oxidative DNA damage, which is what the NISIM is based on. Although ROS can cause damage to DNA, this damage can be countered by DNA repairing processes (Lu et al., 2001). Telomeres, however, are repaired less efficiently than the rest of the genome (Coluzzi et al., 2014).

### Telomere Length and Vagally Mediated Heart Rate Variability in Healthy Participants

Telomeres are stretches of repetitive DNA (TTAGGG or T_2_AG_3_ in vertebrates) and associated proteins that cap the ends of linear chromosomes to protect them from end-to-end fusion by DNA-repair processes. They also protect chromosomal ends from degradation, making them essential in maintaining chromosome and genome stability (d’Adda di Fagagna et al., 2004; Coluzzi et al., 2014). Telomeres interact with telomerase, a ribonucleoprotein complex that further influences chromosome-end integrity by adding telomeric repeats to the chromosome 3′ end (Coluzzi et al., 2014). There is a feed-forward regulatory relationship between telomerase and NFκB, where telomerase regulates NFκB-dependent gene expression, and NFκB transcriptionally regulate telomerase levels. NFκB has an activating role in telomerase expression and activity by up-regulation of human telomerase reverse transcriptase (hTERT), which is an essential catalytic subunit of telomerase in the telomeric repair process (Ghosh et al., 2012; Ramlee et al., 2016). Furthermore, production of H_2_O_2_ and $O_{2}^{-}$ inhibits telomerase activity (Deeb et al., 2013) in addition to causing telomere attrition. TNF-α has also been found to downregulate hTERT gene expression in some cells (Houben et al., 2008).

As previously noted, psychological stress has been linked to cellular senescence (Epel et al., 2004; Sapolsky, 2004). These links include relationships between telomere length and psychological- and oxidative stress (Epel et al., 2004, 2009); greater perceived stress the past month (Mathur et al., 2016); and greater SNS activation and PNS withdrawal after exposure to physical and psychosocial stressors has shown to be associated with shorter telomere length in children (Kroenke et al., 2011). Interestingly, in the latter case, PNS withdrawal was operationalized as decreased respiratory-sinus arrhythmia, which can be quantified as the high frequency indices of HRV that indicate the parasympathetic modulation of heart rate (Berntson et al., 1993). Studies have also shown that telomere length is inversely related to hostility (Brydon et al., 2012), which is inversely related to vagally mediated HRV (Sloan et al., 1994). Moreover, acute mental stress is associated with increased telomerase activity (Epel et al., 2010). If telomere length is linked to stress (Epel et al., 2004; Sapolsky, 2004) then it is reasonable to suggest that it is linked to stress regulation. Few studies have investigated the links between vagally mediated HRV and telomere length and telomerase in healthy participants, specifically. Albeit there being some heterogeneity in the results, they generally seem to point in the same direction.

Men with shorter telomeres and with high telomerase demonstrated blunted post-stress recovery in systolic blood pressure, vagal tone, and monocyte chemoattractant protein-1 (MCP-1), together with reduced responsivity in diastolic blood pressure, heart rate, and cortisol, compared to men with longer telomeres (Zalli et al., 2013). The study also investigated whether levels of IL-6 would be related to telomere length but they did not observe any associations between telomere group and the magnitude of IL-6 responses to the short-term stressor. In a study of individuals aged 23–91 years, elderly people with shorter telomeres had lower vagally mediated HRV compared to people of the same age group with longer telomeres (Streltsova et al., 2017). Telomere length was independently related to vagally mediated HRV when age and gender was controlled for.

Contradictory findings were reported in a study of young healthy women, where the relationship between cortisol, adrenaline, and telomere length as well as telomerase activity and vagal tone indicated by vagally mediated HRV was examined in relation to an acute mental lab stressor. Telomerase activity was related to greater hemodynamic arousal, lower vagal tone, and greater sympathetic reactivity to the acute mental stressor. Cortisol and adrenaline but not vagal tone was found to be associated with telomere length (Epel et al., 2006). Consistent with previous studies, a recent study reported that shorter buccal telomere length was associated with greater cortisol output and reduced vagal tone indicated by vagally mediated HRV in response to a psychosocial stressor. When adjusting for medication use, the relationship between cortisol output and buccal telomere length became non-significant. Telomere length continued to be linked to vagal tone when adjusting for all individual covariates and all covariates simultaneously (Woody et al., 2017b). In a study assessing which of vagally mediated HRV, inflammatory marker CRP, or telomere length would show the strongest association with age, a relationship was found between vagally mediated HRV, CRP, and telomere length, in addition to vagal tone showing the greatest association with age (Perseguini et al., 2015).

These scarce findings must be taken with caution, given the current lack of replications and further corroboration. These preliminary findings indicate that low vagal tone, because of the reduced cholinergic inhibition of cytokine production which in turn increases cytokine induced NFκB activation and subsequent ROS-generation, is linked to reduced telomere length (Zalli et al., 2013; Perseguini et al., 2015; Streltsova et al., 2017; Woody et al., 2017b), as proposed by the NISIM.

### Telomere Length in Vagal Tone-Related Stress Reduction Interventions

A growing body of evidence seems to indicate that stress relaxation techniques (e.g., meditation, mindfulness, yoga) aiming to relieve from psychological and physiological stress, increase vagally mediated state HRV (vagal tone efficiency; Krygier et al., 2013; Azam et al., 2016). The relationship between vagal tone and stress relaxation techniques does seem to depend on which techniques are employed, with those focusing on breathing techniques being most efficient (Lumma et al., 2015). Interestingly, stress relaxation techniques have also been shown to reduce NFκB activity (Dusek et al., 2008; Black et al., 2013). If telomere length is related to psychological stress (Epel et al., 2004, 2009; Sapolsky, 2004) and vagally mediated HRV (Zalli et al., 2013; Woody et al., 2017b), and stress relaxation techniques increase vagal tone efficiency (Krygier et al., 2013; Azam et al., 2016), the NISIM argues for the possibility that interventions applying stress relaxation techniques will influence telomere length and telomerase activity.

Post-retreat telomerase activity was significantly higher in a retreat meditation group compared to matched controls, after controlling for age and body mass index (Jacobs et al., 2011). Another study of regular meditators showed that median relative telomere length was longer in meditation practitioners compared to controls, but that the between group difference was restricted to the female sub-group (Hoge et al., 2013). In an expert meditation group, telomerase activity was reported to be more upregulated compared to a control group and a meditation training group. Within group effects were higher in the expert group and training group at various times of measurements (Bhasin et al., 2013).

In concordance with previous findings, a study investigating the relationship between telomere length and meditation in participants of a 1-month insight meditation retreat showed that telomeres were significantly longer in the retreat group at 3 weeks than at baseline compared to a control group (Conklin et al., 2015). Baseline telomerase activity was lower in regular meditators participating in a meditation retreat compared to women randomized to vacation at the same retreat, but at the day-5 follow up showed a significant increase in telomerase activity that was not seen in the control group (Epel et al., 2016). Further corroboration of the link between stress reduction techniques and telomere integrity was reported in a study that compared expert meditators to matched controls, where expert meditators had significantly longer mean telomere length, and a lower percentage of short telomeres in individual cells compared to the comparison group (Alda et al., 2016). As part of a 12-week yoga and meditation based lifestyle intervention, participants were evaluated at day 0 and week 12 for telomere length and telomerase activity. Mean telomere length was increased at week 12, but not significantly. Mean levels of telomerase was significantly increased at week 12 (Tolahunase et al., 2017). Another study found a significant increase in telomere length in a subset of meditators that partook in their 12-week mediation study (Thimmapuram et al., 2017).

Examinations of post-meditation cellular activity revealed a downregulation of NFκB activation in participants in addition to the reported upregulation of telomerase activity (Bhasin et al., 2013), contradictory to more recent findings, reporting no decrease in NFκB specifically, but suppressions in large inflammatory gene networks in their regular meditator group, and an increase in TNF-α in their vacation group post retreat (Epel et al., 2016). Inflammatory gene expression is regulated by the NFκB pathway (Baker R.G. et al., 2011; Guma et al., 2011) and a growing number of studies have demonstrated that the IKK/NFκB signaling cascade governs gene response to TNF-α in various cells (Krappmann et al., 2004; Bhatnagar et al., 2010) with secondary transcriptional responses involving other regulators being submitted to NFκB control (Kempe et al., 2005). If the observed differences in TNF-α levels were due to meditation, then it is very possible that there was a difference in subsequent NFκB activity that could have affected the differences in telomerase activity (Epel et al., 2016). Furthermore, attenuations in cellular stressors were observed among participants in the 12-week yoga and meditation intervention study, with mean levels of 8-OH-dGua, ROS, and IL-6 being decreased among meditators post intervention (Tolahunase et al., 2017).

Stress relaxation techniques that increase vagal tone efficiency (Krygier et al., 2013; Lumma et al., 2015; Azam et al., 2016) seem to have an effect on telomere integrity (Jacobs et al., 2011; Bhasin et al., 2013; Hoge et al., 2013; Conklin et al., 2015; Alda et al., 2016; Epel et al., 2016; Thimmapuram et al., 2017; Tolahunase et al., 2017), which could perhaps be attributed to reduced inflammatory cytokine production due to increased cholinergic stimulation of macrophages, and therefore reduced NFκB activity, which is what the NISIM argues.

## The DNA Damage Response

The evidence seems to indicate that there is a link between prefrontally modulated vagal activity and telomere length. Loss of telomeric repeat sequences or deficiencies in telomeric proteins can result in dysfunctional telomeres and chromosome instability (Murnane, 2012). Telomeres can lose a vast number of base pairs before it is rendered dysfunctional, yet one dysfunctional telomere is enough to trigger a classical DDR which enables cells to sense damaged DNA (d’Adda di Fagagna et al., 2003; Takai et al., 2003; Herbig et al., 2004). If possible, the cell responds to DDR by arresting cell-cycle progression and repairing damage (Martens et al., 2000; Hemann et al., 2001). The growth arrest is established and maintained by the p53 and p16-pRB tumor suppressor pathways. These pathways can halt cell-cycle progression both independently and through interaction (Olsen et al., 2002; Michaloglou et al., 2005). The p53 pathway is the primary senescence inducer with telomere damage (Sherr and McCormick, 2002), although a lot of different stressors can activate the p53 pathway. Once the p53 protein is activated it initiates a transcriptional program that reflects the stress signal, the protein modifications and the proteins associated with the p53 protein. The p53 then binds to a specific DNA sequence, initiating one of three programs resulting in arrested growth, cellular senescence, or apoptosis. The program that is selected depends on the stress signal (Harris and Levine, 2005).

In sum, as there seem to be specific links between parasympathetic dysfunction, cytokine production, NFκB activation, and telomeric DNA damage, it supports the case for the NISIM.

## Applying the Model: Neurodegenerative Disease and Cancer

**FIGURE 2:**
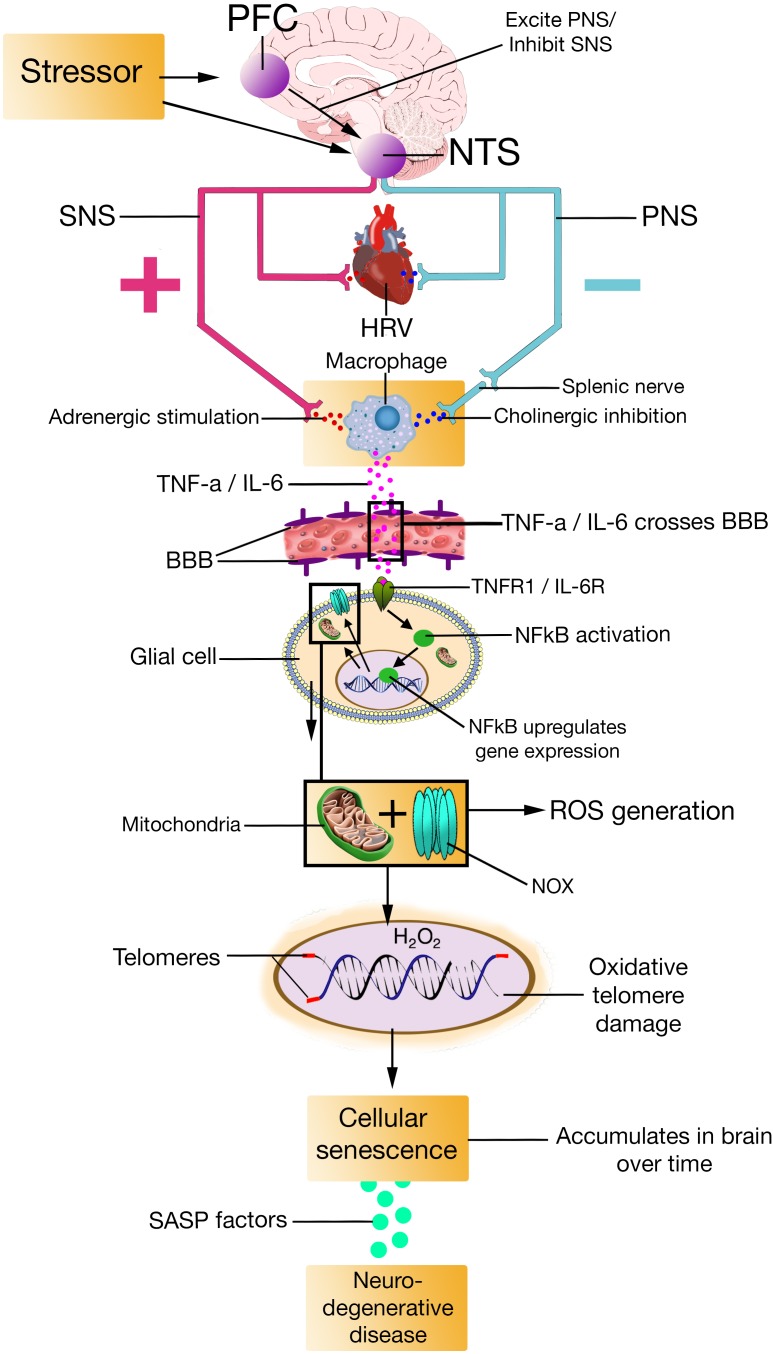
PFC, prefrontal cortex; NTS, nucleus of the tractus solitarius; SNS, sympathetic nervous system; PNS, parasympathetic nervous system; HRV, heart rate variability; TNF-a, Tumor Necrosis Factor-alpha; IL-6, interleukin-6; TNFR1, Tumor Necrosis Factor-alpha Receptor 1; IL-6R, interleukin-6 receptor; BBB, blood-brain barrier; NFkB, Nuclear factor kappa light chain enhancer of activated B cells; H2O2, hydrogen peroxide; ROS, reactive oxygen species; NOX, nicotinamide adenine dinucleotide phosphate oxidase; SASP, senescence-associated secretory phenotype.

**FIGURE 3:**
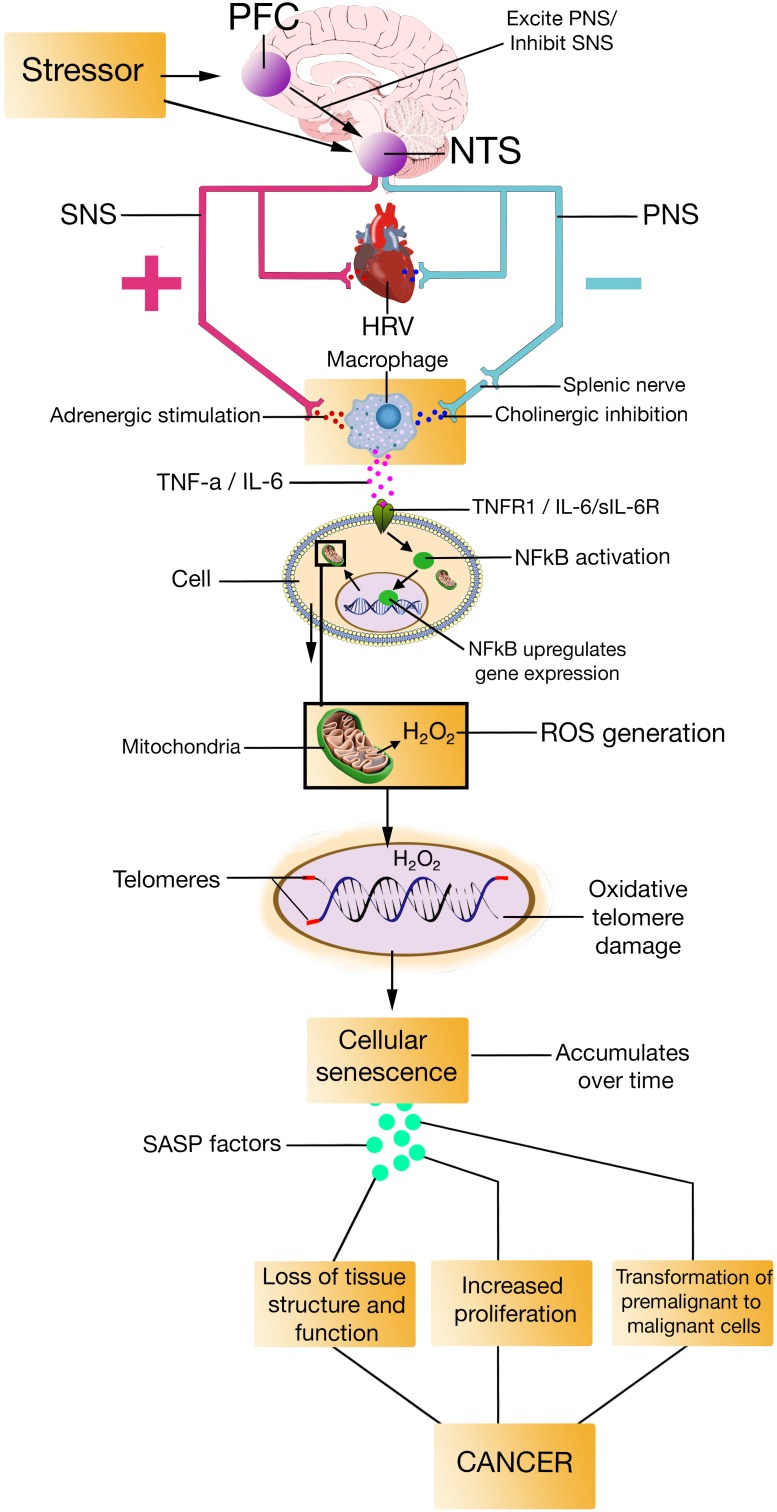
PFC, prefrontal cortex; NTS, nucleus of the tractus solitarius; SNS, sympathetic nervous system; PNS, parasympathetic nervous system; HRV, heart rate variability; TNF-a, Tumor Necrosis Factor-alpha; IL-6, interleukin-6; TNFR1, Tumor Necrosis Factor-alpha Receptor 1; sIL-6R, soluble interleukin-6 receptor; NFkB, Nuclear factor kappa light chain enhancer of activated B cells; H2O2, hydrogen peroxide; ROS, reactive oxygen species; SASP, senescence-associated secretory phenotype.

The SASP is primarily a DDR, meaning that it occurs in cells that senesce in response to DNA damage (Rodier et al., 2009; Campisi, 2013), which – according to the model – would be the case for individuals with low prefrontally modulated vagal activity. The SASP is also dependent on the activation of other signaling pathways, including p38MAPK, and mechanistic target of rapamycin (Coppé et al., 2008). The factors that the SASP secrete include proteases, cytokines such as TNF-α, IL-6, and IL-8, growth factors such as insulin-like growth factor binding proteins (IGFBPs), among others (Bhat et al., 2012; Tchkonia et al., 2013). Most SASP factors are up-regulated at the mRNA level. The mRNA up-regulation of SASP factors are in part due to increased activity of transcription factor proteins like NFκB and CCAAT/enhancer binding protein (Coppé et al., 2008; Freund et al., 2010). By secreting SASP factors, the SASP can trigger immune surveillance of senescent cells (Xue et al., 2007), enforce cell cycle arrest (Acosta et al., 2008; Bartek et al., 2008), and alter tissue microenvironments (Tchkonia et al., 2013). Evidence also suggest that the SASP can induce paracrine senescence in normal cells (Kuilman et al., 2008; Yu et al., 2017) via the SASP proteins IGFBP7 (Wajapeyee et al., 2008) and IL-6 (Acosta et al., 2013), which goes further in explaining how the SASP alter tissue microenvironments (González-Puertos et al., 2015). The NISIM, by integrating the SASP, could perhaps help elucidate some of the antecedent aspects regarding the induction of age-related pathologies (**Figures 2**, **3**).

### Senescence and the Brain: Neurodegenerative Diseases

Mitotic glial cells are the most likely prospects for cellular senescence in the brain. This is because neurons are generally incapable of cell division, thus making them less likely candidates for carcinogenesis (Frade and Ovejero-Benito, 2015). Several recent studies suggest that senescent cells are detectable in the mammalian brain (Chinta et al., 2015). A question of interest is how prefrontally modulated vagal tone and subsequent peripheral production of cytokines would relate to brain cell senescence. This can perhaps be answered by a recent article where Maletic and Raison (2014) proposed five pathways for transfer of peripheral inflammatory signals to the brain: Leaky areas in the blood-brain barrier (BBB) makes it possible for circulating cytokines to enter the brain. Afferent vagal fibers convey peripheral inflammatory signals to their nuclei. Certain BBB cells have systems for active transport of peripheral cytokines into the brain. Peripheral immune cells can migrate into the brain and release inflammatory mediators by means of *trans*-vessel translocation. The endothelial cells that makes up the BBB release inflammatory signals.

Blood-brain barrier transportation systems for TNF-α and IL-6 are very well documented, especially in the case of TNF-α (Pan et al., 2011). Furthermore, several studies suggest that the vascular permeability of the BBB increase with age, making it easier for proteins to enter the brain (Zeevi et al., 2010). TNF-α has been demonstrated to increase BBB permeability by causing disruption of tight junctions and endothelial cell shrinkage (Lv et al., 2010). Interestingly, H_2_O_2_ can increase transendothelial permeability, allowing for macromolecules to cross vascular walls (Shasby et al., 1985; Wilson et al., 1990). If either the cytokines that are observed in people with low vagal tone or equivalent cytokines that are released by senescent cells in the periphery are transported into the brain, they could possibly induce telomere damage in glial cells and cause senescence.

Human astrocytes are mitotic glial cells found throughout the central nervous system (CNS), and they are essential for maintaining brain homeostasis (Chen and Swanson, 2003; Sofroniew and Vinters, 2010). There is currently a growing body of literature describing senescent astrocytes both *in vivo* and *in vitro* (Nichols et al., 1993; Pertusa et al., 2007; Turnquist et al., 2016). TNF-α and IL-6 has been reported to induce both senescence and chromosomal instability in certain cell types (Beyne-Rauzy et al., 2004; Acosta et al., 2013), and astrocytes express receptors for both TNF-α and IL-6 (McCoy and Tansey, 2008; Erta et al., 2012). TNF-α stimulation of astrocytes cause a robust increase in NFκB translocation to the nuclei subsequently increasing mitochondrial respiration. This effect increases with age. Aging astrocytes also display an increase in NOX2-induced H_2_O_2_ (Jiang and Cadenas, 2014). NFκB is a potent inducer of NOX such as NOX2 (Anrather et al., 2006; Morgan and Liu, 2011) and TNF-α can induce NOX2 activity in a NFκB-dependent manner (Li et al., 2009). IL-6 also seem to induce ROS via NOX2 (Behrens et al., 2008; Wang et al., 2016). Astrocytes treated with TNF-α also show an upregulation of p38MAPK (Thompson and Van Eldik, 2009) which contributes to the upregulation of SASP factors in cellular senescence (Coppé et al., 2008; Freund et al., 2010). IL-6 receptor stimulation of astrocytes also seems to activate NFκB and p38MAPK (Ma et al., 2010). After exposure to exogenous H_2_O_2_, cultured astrocytes display several characteristics of cellular senescence, such as arrested growth and increased expression of p21 and p16^INK4a^. They also seem to be more sensitive to OS than fibroblasts, suggesting that OS-induced senescence is more pronounced in the brain compared to other tissues (Bitto et al., 2010). TNF-α has been shown to induce ROS through the NOX system in isolated microglia (Mander et al., 2006).

Dysfunctional astrocytes and microglia are associated with several age-related neurodegenerative diseases (Chen and Swanson, 2003; Benarroch, 2005; Chinta et al., 2015), and several lines of data suggest that age related neurodegenerative diseases are accompanied by an increase in SASP-expressing senescent cells of non-neuronal origin (Chinta et al., 2015). Chronic age-related neurodegenerative diseases are also associated with increases in SASP factors, such as IL-6 and IL-1β (Bachstetter et al., 2011). The relationship between psychological stress and onset of neurodegenerative disease is not an unexplored one. For example, links between stress and Parkinson disease (PD) have been proposed (Djamshidian and Lees, 2014; Austin et al., 2016), and refuted (Clark et al., 2013). However, a prospective study that examined vagal tone indicated by vagally mediated HRV in relation to risk of PD showed that low vagal tone was associated with higher risk of PD 18 years later (Alonso et al., 2015). Chronic psychosocial stress at work, characterized by high job demand and low job control, is associated with increased dementia and Alzheimer’s disease (AD) risk in late life (Wang et al., 2012) but not stressful life events (Sundström et al., 2014). According to the ‘perseverative cognition hypothesis’ (Brosschot et al., 2005), will stress only be detrimental to physical health if the cognitive representation of the stress-related content is chronically or repeatedly activated, such as through worry or rumination. Work-related rumination has an inverse relationship with vagal tone (Cropley et al., 2017), and high job demand and low job control is associated with reductions in both vagally and sympathetically mediated indices of HRV during 24 h monitoring of resident physicians (Hernández-Gaytan et al., 2013). Furthermore, several studies, rather consistently, find that parasympathetic dysfunction, indicated by reduced vagally mediated HRV is associated with lower cognitive performance in people aged 50 years and older (Kim et al., 2006; Al Hazzouri et al., 2017), although Britton et al. (2008) found no such association. Parasympathetic dysfunction, indicated by low vagally mediated HRV is correlated with degree of cognitive impairment in AD (Zulli et al., 2006), and there also seem to be a relationship between management of cardiovascular risk factors and reducing risk of AD-related cognitive decline (Santos et al., 2017). Although neurodegenerative diseases are notoriously complex and multi-causal, autonomic imbalance characterized by relative sympathetic over parasympathetic dominance do seem to play some part in its development.

### Cellular Senescence and Cancer

Although being a tumor-suppressive program, the SASP sometimes promotes cancer in nearby cells (Campisi, 2013; Tchkonia et al., 2013). For example, the SASP is suspected to cause the loss of tissue structure and function observed in aging by creating pro-inflammatory milieus (Chinta et al., 2015) which is a prerequisite for cancer induction and progression (Bissel et al., 2005). The pro-inflammatory milieus can in large be attributed to the increased NFκB activation (Karin, 2009; Lawrence, 2009). Further promotion of cancer is usually achieved by driving proliferation and metastasis in cells that are premalignant (Rao and Jackson, 2016), as well as ensuring survival, growth and vascularization in full blown malignancy (Chinta et al., 2015). Several studies indicate that SASP factors such as IGFBPs can display both tumor-suppressive and oncogenic effects. Although the evidence for oncogenic effects is mixed for several IGFBPs (Xu et al., 2010; Baxter, 2014), the emergence of a growth-promoting role in tumor systems is most evident for IGFBP-2 (Dunlap et al., 2007). Over expressed or exogenous IGFBP-2 increase cancer cell growth as well as potential for metastasis (Fukushima et al., 2007). Moreover, a recent study reported that IL-6 and IL-8 are necessary and sufficient to increase tumor cell migration through a synergistic paracrine signaling pathway (Jayatilaka et al., 2017), and suppression of these SASP factors in senescent cells decrease their ability to promote proliferation in cancer cells (Hou et al., 2017). There is also evidence that TNF-α has a direct role in cancer cell survival (Kottke et al., 2017). So, if the SASP do not induce cancer, it at least contributes to cancer growth and metastasis.

There are several cellular events that have the possibility of being tumorigenic, including genetic mutations (Blackadar, 2016). Studies investigating psychological stress as a definite contributor to risk for cancer occurrence are by and large inconclusive. For example, low socioeconomic status, a known chronic stress factor, is linked to increased cancer incidence for some cancer types, but not all (Clegg et al., 2009). Several potential mechanisms for stress-related cancer induction have been proposed, usually linking stress- and depression related CNS activity to immune responses and HPA-axis regulated hormonal secretion (Soung and Kim, 2015; Shin et al., 2016). In support of the proposed links between psychological stress and cancer, a recently published prospective cohort study found that adolescent psychological stress resilience was associated with adult cancer occurrence, even after adjusting for socioeconomic circumstances in childhood, and cognitive and physical fitness (Kennedy et al., 2017). Vagal tone is thought to reflect individual stress resilience (Segerstrom and Nes, 2007; McCraty and Shaffer, 2015) and several of the characteristics Kennedy et al. (2017) used to estimate psychological stress resilience are associated with vagal tone, including persistence (Segerstrom and Nes, 2007; Reynard et al., 2011), emotional stability (Koval et al., 2013), social coherence (McCraty, 2017), and adolescent antisocial behavior (Mezzacappa et al., 1997). Moreover, vagal tone indicated by vagally mediated HRV predicts capability of regulating arousal during prolonged stressors (Hildebrandt et al., 2016) which links stress regulation capacity to cancer occurrence in adulthood.

## Predictions and Conclusion

**FIGURE 4:**
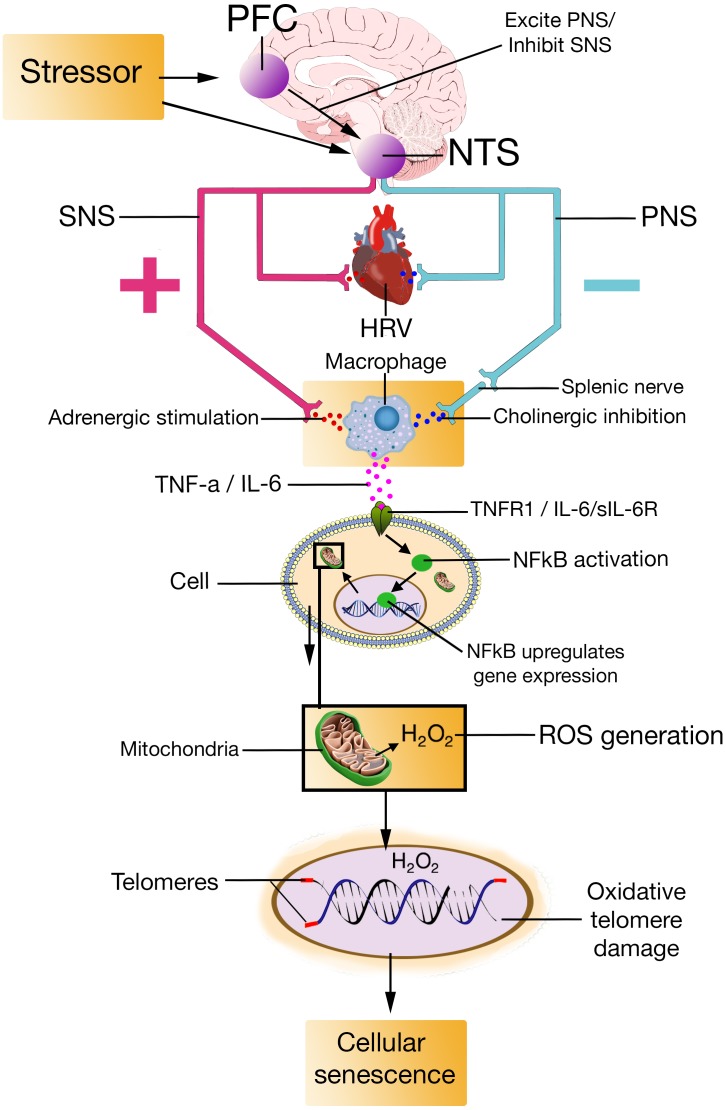
PFC, prefrontal cortex; NTS, nucleus of the tractus solitarius; SNS, sympathetic nervous system; PNS, parasympathetic nervous system; HRV, heart rate variability; TNF-a, Tumor Necrosis Factor-alpha; IL-6, interleukin-6; TNFR1, Tumor Necrosis Factor-alpha Receptor 1; sIL-6R, soluble interleukin-6 receptor; NFkB, Nuclear factor kappa light chain enhancer of activated B cells; H2O2, hydrogen peroxide; ROS, reactive oxygen species.

In this paper, we proposed the NISIM suggesting how prefrontal stress regulation as indexed by HRV relates to cellular senescence (**Figure 4**). A growing body of research converges on inadequate vagus nerve activity as a unifying factor in several clinical conditions characterized by increased inflammation and oxidative stress (De Couck et al., 2012). The NISIM not only points to vagal activity as a unifying factor in the conditions discussed in this paper, but also propose causal mechanisms based on established relationships between prefrontally modulated, and vagally mediated, cortical influences on the ANS, and how ANS-immune system interactions result in the subsequent cytokine production, cytokine-activated NFκB-induced ROS generation, and ROS-induced telomere shortening that induce senescence. Previous models have proposed links between psychological stress and telomere length (Sapolsky, 2004; Epel et al., 2009), but as far as we know, the NISIM is the first model outlining specific mechanisms for how these phenomena are related. It is important to specify that while the NISIM attempts to provide a plausible explanation for how psychological stress relates to cellular aging, it is not an attempt at implicating low prefrontal activity and subsequent parasympathetic dysfunction as a primary driver of cellular senescence. Nor does the model claim that age-associated inflammation or senescence is primarily driven by immune-competent cells. Cellular senescence can happen to any mitotic cell in response to a variety of cellular stressors (Campisi, 2001, 2003); the claim made by the NISIM is merely that low trait prefrontal activity speeds up the accumulation of senescent cells.

A couple of predictions can be made from the NISIM. First, individual differences in accumulation of senescent cells should in part be related to vagally mediated HRV because of the mechanisms discussed above, with prefrontal input to the ANS and subsequent autonomic output to cytokine producing organs having an effect on telomere length via IL-6 and TNF-α. Second, vagal tone should be related to NFκB activity and mitochondrial respiration. This might have several implications for telomerase activity; since NFκB upregulates telomerase (Ghosh et al., 2012) and both ROS and TNF-α can suppress telomerase activity (Houben et al., 2008; Deeb et al., 2013), then the effect of vagal activity on telomerase activity might depend on degree of NFκB activation. Third, if parasympathetic dysfunction induce inflammatory responses which in turn cause ROS-induced damage to DNA, then vagally mediated HRV should be related to urinary levels of 8-OH-dGua in otherwise healthy populations. It is possible that other inflammatory cytokines can be implicated in the mechanisms proposed by this model, but the evidence for that is currently lacking.

This hypothetical argumentation has some limitations. The attempt to use the NISIM in explaining senescence in CNS tissues, and subsequent development of age-related neurodegenerative diseases, relies on the extent cytokines of peripheral origin are transported across the BBB. Although the evidence for such transportation systems are numerous (Pan et al., 2011), it does not necessarily equate to the effects discussed in this paper. However, as astrocytes are more sensitive to oxidative stress than other cell types (Bitto et al., 2010), and since they display an increase in NFκB activity in response to TNF-α (Jiang and Cadenas, 2014), in addition to that TNF-α also induce ROS via NOX systems in microglia (Mander et al., 2006), some support is provided. However, TNF-α does not seem to induce ROS production via NOX2 in RPE cell cultures but via mitochondrial respiration (Yang et al., 2007), which might indicate that the NOX-dependent pathway has limited generalizability. The fact that neurodegenerative diseases are linked to SASP expressing glial cells (Bachstetter et al., 2011; Chinta et al., 2015), that vagal tone is associated with PD (Alonso et al., 2015), symptom severity in AD (Zulli et al., 2006), and cognitive decline in adults (Kim et al., 2006; Al Hazzouri et al., 2017), and that psychosocial stress predicts late AD (Wang et al., 2012) provide support for the vagal tone-senescence-neurodegenerative disease link. Experimental verification of vagal tone in relation to senescence induction in astrocytes would be necessary to fully corroborate this link, although such investigations might be limited by the current methodologies for detecting senescent cells (Salmonowicz and Passos, 2017). There is also the question if senescent cells cause cancer or simply drive growth and metastasis, the latter case being the more plausible (Collado et al., 2007; Campisi, 2013). The link between adolescent psychological stress resilience and cancer occurrence in adulthood (Kennedy et al., 2017) does seem to support the hypothesis that stress and therefore stress regulation is linked to cancer, and the link between prefrontally modulated vagal activity, senescence and cancer seems plausible. Prospective studies on vagal tone and cancer occurrence is needed.

Another limitation would be the samples that the vagal tone-cytokine link is based on. Future studies should be mindful of confounding variables such as time frame for measuring cardiac activity and when blood samples for cytokine analysis are drawn. Vagal activity does not remain constant throughout the day, so correlating it to inflammation could be limited to time frame of the analysis (Haensel et al., 2008; Papaioannou et al., 2013). For instance, nocturnal vagally mediated HRV is associated with morning levels of IL-6 and CRP in individuals without medical conditions (Bell et al., 2017). Depression is another major confounder (Kop et al., 2010; Felger and Lotrich, 2013), so the degree to which participants have been screened for depressive symptoms, or people with depression is included in the sample could affect the level of cytokines that are observed. One study included depressed people in their sample (Lampert et al., 2008), and three studies did not screen for previous or current depression (Sloan et al., 2007; von Känel et al., 2007; Woody et al., 2017a). Five studies did not exclude smokers (Owen and Steptoe, 2003; Sloan et al., 2007; von Känel et al., 2007; Lampert et al., 2008; Weber et al., 2010), and at least two studies included people with hypertension and diabetes (Sloan et al., 2007; Lampert et al., 2008). Moreover, the high frequency power in the study by von Känel et al. (2007) explained only a limited portion of the variance in IL-6 and soluble tissue factor.

It has also been suggested that sympathetic activity can both inhibit and induce cytokine production, whilst there is uncertainty if vagally mediated HRV reflects vagal influence on the spleen and other major cytokine producing organs (Haensel et al., 2008; Papaioannou et al., 2013), which could be the reason why one of the studies had negative results (Owen and Steptoe, 2003). Heterogeneity in the results could possibly be owed to differences in stressor task paradigms, such as the intensity of the stressor applied. Other methodological issues like technical malfunction could also affect results; Owen and Steptoe (2003) reported that vagally mediated HRV was only obtained from 159 of the 211 participants in their study, due to equipment breakdown, which may have contributed to their negative results. The overall evidence, albeit mixed, do seem to corroborate the HRV-cytokine link for IL-6 and TNF-α.

Finally, the studies on telomere length and vagal tone are scarce, only five studies were examined in this paper (Epel et al., 2006; Zalli et al., 2013; Perseguini et al., 2015; Streltsova et al., 2017; Woody et al., 2017b), and one of the studies did not find any association (Epel et al., 2006). Further replication will be needed. It is also important to note that one of the studies assessing the association between vagally mediated HRV and telomere length also assessed whether IL-6 magnitude would be associated with telomere length, but they did not find any association (Zalli et al., 2013). The link between meditation and increases in vagal tone efficiency (Krygier et al., 2013; Azam et al., 2016), and meditation and increased telomere length and telomerase activity (Jacobs et al., 2011; Bhasin et al., 2013; Hoge et al., 2013; Conklin et al., 2015; Alda et al., 2016; Epel et al., 2016; Thimmapuram et al., 2017; Tolahunase et al., 2017) do provide some additional support for the vagal tone-telomere length link.

Several research designs could be employed to falsify the NISIM, the key being to establish whether baseline prefrontal activity can be implicated as a causal contributor to some of the individual differences in accumulated SASP factor-expressing senescent cells, and consequently, stress-related health span. The model is currently based on converging and correlational evidence; however, longitudinal designs are needed. It might be necessary to use both human participants and animal models. For instance, several studies comparing the rat medial PFC to that of primates seem to indicate that there is overlap in the functioning of the anterior cingulate cortex (ACC) and the dorsolateral PFC, at least in rudimentary functioning (Seamans et al., 2008). Both meta-analysis of brain activity and studies on brain structure in relation to vagally mediated HRV has implicated the ACC as an associated neural component (Thayer et al., 2012; Yoo et al., 2018). Moreover, individual differences in vagally mediated HRV in rats is associated with flexible adaptation to various stressors (Carnevali and Sgoifo, 2014). Although it is not apparent that efferent medial PFC activity in rats modulate vagal activity in the same way it does in humans, one possible way of falsifying the NISIM is through investigating whether induction of prefrontal lesions to the rat medial PFC would result in increased accumulation of senescent cells compared to healthy controls and models with lesions to other brain areas. It might, however, be necessary to first establish whether prefrontal lesions will have an effect on either vagally mediated HRV or vagal input to the spleen in order to establish whether the rat is a valid animal model for falsifying the NISIM. Other possible research designs in animal models could be examining the relationship between vagal activity and markers of NFκB activity, mitochondrial respiration, and NOX-2 activity in various tissues.

Current limitations regarding *in vivo* examination of senescent cells makes detection in humans a somewhat difficult task. First and foremost, replication of previous studies assessing the relationship between vagally mediated HRV and telomere length and cytokine levels in healthy participants is necessary. Studies should also investigate whether the association between vagal tone and telomere length is mediated by IL-6 and TNF-α. It would be interesting to see if trait vagally mediated HRV is associated with repeated measures of urinary of 8-OH-dGua as this marker is a product of oxidative damage to DNA (Kasai et al., 2008; Valavanidis et al., 2009; Coluzzi et al., 2014). To validate the practical applicability of the NISIM, prospective studies in humans should assess whether markers of trait vagal tone is associated with Alzheimer’s and cancer onset. Longitudinal studies applying brain imaging techniques in healthy individuals should investigate whether structure and metabolic activity in the prefrontal areas discussed by Thayer et al. (2012) and Yoo et al. (2018) is associated with repeated measures of vagally mediated HRV, telomere length, increases in age-associated inflammation, and disease onset. In the case of cancer, research should look for associations between vagal tone and number of senescent cells in tumor biopsies. Vagal-activating therapies could also be applied to longitudinal research designs to assess whether they will be associated with reduced urinary 8-OH-dGua, longer telomeres, and reduced serum levels of inflammatory cytokines compared to control groups.

An important aspect when using vagally mediated HRV as a trait indicator of prefrontal input to the ANS is to apply multiple measurement points when recording of inter-beat intervals. Bertsch and colleagues suggested that 40% of the variance of a single HRV measurement can be attributed to effects of the situation; at least two measurements are recommended when using HRV as a consistent biomarker or trait (Bertsch et al., 2012). A time-consuming endeavor, however, it is necessary in these kinds of studies. Alternative measures of parasympathetic dysfunction should be considered; heart rate recovery has been independently associated with inflammation (Ackland et al., 2018) and could thus serve as viable indicator of vagal activity.

A second important issue lies in the difficulty of detecting senescent cells; universal biomarkers that unambiguously distinguish between senescent and non-senescent cells are currently not reported in the literature. Senescent cells are hard to detect for several reasons: several of the genetic changes that occurs in cells undergoing senescence is not exclusive to senescent cells; senescence is a multifactorial process with phenotypic changes occurring at different times; the phenotypic profile of senescent cells varies according to stimuli and cell type, and; senescent cells have different roles that varies according to physiological context (van Deursen, 2014). The most frequently used method of detecting senescent cells is through detection of senescence-associated β-galactosidase (SA-β-Gal) activity at pH 6 (Dimri et al., 1995). However, it has been suggested that SA-β-Gal staining may not be exclusive to senescent cells (Cristofalo, 2005). This can potentially increase false positives, and multimarker approaches has received favorable appraise (Narita et al., 2003; Coppé et al., 2008; Shimi et al., 2011; Hewitt et al., 2012). A new promising staining method called GL13 was recently proposed based on detection of lipofuscin using an analog of Sudan Black B histochemical dye coupled with biotin (Evangelou et al., 2017). This method is potentially more sensitive than SA-β-Gal staining due to the biotin coupling allowing it to be detected using anti-biotin antibodies and identified using both microscopy and flow cytometry (Evangelou et al., 2017), although the potential for detecting false positives has not been completely abolished (Salmonowicz and Passos, 2017). One novel multimarker approach that attempts to reduce protocol length while at the same time allowing for quantification suggest taking advantage of newly discovered extracellular plasma membrane markers for fast detection of senescent cells (Althubiti and Macip, 2017). Biran and colleagues developed a method for detection and quantification of senescent cells in tumors, fibrotic tissues, and aged tissue (Biran et al., 2017). The method is a single-cell based approach applying different senescence-markers while combining flow cytometry with high-content image analysis.

A third issue is owed to the fact that the relationship between vagal tone, cytokine levels, and ROS is not linear. In addition to cytokines being capable of inducing production of ROS (Schreck and Bauerle, 1994), imbalance between ROS and anti-oxidants can induce production of pro-inflammatory cytokines such as TNF-α, IL-1, and IL-8 which can affect HRV by entering the peripheral circulatory system (Donaldson et al., 2001; Nelin et al., 2012). Preexisting OS and inflammation can increase production of pro-inflammatory cytokines (Stringer and Kobzik, 1998; Donaldson et al., 2001) resulting in autonomic imbalance and reduced parasympathetic tone (Aronson et al., 2001). Furthermore, oxidative stress and systemic inflammation has been identified as modifiers of cardiac autonomic responses to particulate air pollution, where levels of urinary 8-OH-dGua, as well as CRP predicts decreases in vagally mediated HRV in repeated measures (Lee et al., 2014). There is also evidence that increases in non-enzymatic anti-oxidant defenses that scavenges H_2_O_2_ and $O_{2}^{-}$ increase vagal tone (Campos et al., 2014). This shows that low vagally mediated HRV may in some cases be induced by oxidative damage because of exposure to environmental pollutants, which could be a possible confounder in future studies. If participants are tested right after arriving at the testing sites, then relationships between vagally mediated HRV and cytokine levels could very well be due to traffic exposure that day (Adar et al., 2007). An overnight stay at the testing site might be a safe option.

### Conclusion

We conclude that the NISIM is useful in explaining the stress-related mechanisms behind the antecedents to cellular senescence, which furthers the understanding of how individual differences in psychological stress regulation capacity is associated with telomere length. This in turn provides an account for some of the individual differences in senescence-related health span. As the literature is converging on the importance of vagal function in pathological conditions (De Couck et al., 2012), it is possible that the NISIM can be extended to other conditions associated with reduced parasympathetic activity and biomarkers of cellular senescence. Current limitations arise from the fact that the NISIM is largely based on correlational studies and converging evidence. Future research should apply longitudinal studies of animal models with PFC lesions to investigate whether it will be associated with parasympathetic activity and accumulation of senescent cells. Short term human studies should replicate previous research while employing stricter criteria for inclusion; multiple measurement points for vagally mediated HRV; and conditions controlling for confounding directionality in associations between vagal tone, cytokine levels, and ROS. Longitudinal human studies should focus on links between PFC structure and activity, vagal activity, changes in telomere length, increases in age-associated inflammation, and disease onset. Methods for detection of senescent cells in biopsies of pathological tissues such as tumors should be combined with longitudinal studies. Lastly, it is important to note that the stress response and its regulation should be considered as one of many modulators of autonomic balance, and thus, the NISIM should be interpreted accordingly.

## Author Contributions

TA: main developer of the proposed model, reviewed the literature cited in the manuscript, and wrote most of it. RL: expert on cognition, provided input to the theoretical framework, and suggestions for revision of the manuscript. SS: expert on heart rate variability, provided input to the theoretical framework, and suggestions for revision of the manuscript.

## Conflict of Interest Statement

The authors declare that the research was conducted in the absence of any commercial or financial relationships that could be construed as a potential conflict of interest.
